# Supplementary material for: Increasing calling accuracy, coverage, and read-depth in sequence data by the use of haplotype blocks
Source: PLoS Genet. 2021 Dec 23;17(12):e1009944. doi: 10.1371/journal.pgen.1009944 (PMC8699914; doi:10.1371/journal.pgen.1009944)
Supplement: S1 Table — (DOCX) [file pgen.1009944.s001.docx]

S1 Table. Predictive ability for the nine maize traits depending on the genotype data used. Details on the individual traits and growing stages (v3-final) can be found in Hölker et al. [1].

|  | HB-seq | HB-seq (overlap) | Array data |
| --- | --- | --- | --- |
| Early vigor (v3) | 0.3795 | 0.3863 | 0.3856 |
| Early vigor (v4) | 0.3851 | 0.3912 | 0.3898 |
| Early vigor (v6) | 0.4651 | 0.4674 | 0.4664 |
| Plant height (v4) | 0.5192 | 0.5229 | 0.5217 |
| Plant height (v6) | 0.5607 | 0.5635 | 0.5632 |
| Plant height (final) | 0.7081 | 0.7086 | 0.7078 |
| Days to silking | 0.5377 | 0.5387 | 0.5387 |
| Days to tassel | 0.4897 | 0.4949 | 0.4905 |
| Root lodging | 0.5714 | 0.5770 | 0.5754 |

**References**

1. Hölker AC, Mayer M, Presterl T, Bolduan T, Bauer E, Ordas B, et al. European maize landraces made accessible for plant breeding and genome-based studies. Theoretical and Applied Genetics. 2019; p. 1–13.
