# Supplementary material for: Increasing calling accuracy, coverage, and read-depth in sequence data by the use of haplotype blocks
Source: PLoS Genet. 2021 Dec 23;17(12):e1009944. doi: 10.1371/journal.pgen.1009944 (PMC8699914; doi:10.1371/journal.pgen.1009944)
Supplement: S2 Table — (DOCX) [file pgen.1009944.s002.docx]

S2 Table. Number of true underlying QTL identified depending on the false discovery rate (FDR).

| FDR | Array (600k) | HB-seq | HB-array | BEAGLE | HB-seq (overlap) | HB-seq (large) | Array (50k) | Array (10k) |
| --- | --- | --- | --- | --- | --- | --- | --- | --- |
| 0.40 | 0.21 | 0.24 | 0.28 | --- | 0.18 | 0.22 | 0.13 | --- |
| 0.45 | 0.49 | 0.48 | 0.56 | 0.46 | 0.41 | 0.51 | 0.25 | 0.03 |
| 0.50 | 0.99 | 0.87 | 1.00 | 0.91 | 0.76 | 0.83 | 0.61 | 0.06 |
| 0.55 | 1.51 | 1.46 | 1.62 | 1.53 | 1.35 | 1.40 | 1.11 | 0.26 |
| 0.60 | 2.14 | 2.11 | 2.27 | 2.19 | 1.95 | 2.02 | 1.70 | 0.72 |
| 0.65 | 2.83 | 2.73 | 2.91 | 2.83 | 2.59 | 2.62 | 2.34 | 1.20 |
| 0.70 | 3.55 | 3.44 | 3.62 | 3.57 | 3.29 | 3.29 | 3.06 | 1.72 |
| 0.75 | 4.33 | 4.22 | 4.38 | 4.37 | 4.05 | 4.05 | 3.89 | --- |
