# Supplementary material for: Increasing calling accuracy, coverage, and read-depth in sequence data by the use of haplotype blocks
Source: PLoS Genet. 2021 Dec 23;17(12):e1009944. doi: 10.1371/journal.pgen.1009944 (PMC8699914; doi:10.1371/journal.pgen.1009944)
Supplement: Attachment — Submitted filename: Response_to_Review_Hbimpute.pdf [file pgen.1009944.s005.pdf]

Dear Dr Pook,

Thank you very much for submitting your Research Article entitled 'Increasing calling accuracy, coverage, and read depth in sequence data by the use of haplotype blocks' to PLOS Genetics.

The manuscript was fully evaluated at the editorial level and by independent peer reviewers. The reviewers appreciated the attention to an important problem, but raised some substantial concerns about the current manuscript. Based on the reviews, we will not be able to accept this version of the manuscript, but we would be willing to review a much-revised version. We cannot, of course, promise publication at that time.

Should you decide to revise the manuscript for further consideration here, your revisions should address all of the specific points made by each reviewer. In particular, you should more thoroughly compare to both BEAGLEv4.1 and STITCH, where some advantage over these existing approaches should be demonstrated. We will also require a detailed list of your responses to the review comments and a description of the changes you have made in the manuscript.

Dear PLoS Genetics Editorial team, Dear Reviewers,

Please find a new version of our manuscript “Increasing calling accuracy, coverage, and read-depth in sequence data by the use of haplotype blocks”. As suggested by both the editor and reviewers, we added a more thorough comparison with the existing state-of-the-art method and thus further highlight the high imputation accuracy obtained with our approach.

You can find detailed comments on all points made by the reviewers below. Please also find the diff-files to make it easier to spot changes made to the manuscript. In particular, note that reported imputing error rates are slightly different as markers that were fixated when using FreeBayes and excluded from the analysis were now considered in the genotype calling of STITCH. Actual differences to the underlying software code from the previous iteration of the manuscript are minimal.

Reviewer's Responses to Questions

Comments to the Authors:

Please note here if the review is uploaded as an attachment.

Reviewer #1: The authors present a method called HBImpute for estimating genotypes from low coverage (e.g. 0.5x) sequence data. The method is designed for the special case of samples with homozygous genotypes (double haploid lines) that arise in plant breeding. The method identifies haplotype blocks and clusters sequence reads from identical by descent haplotypes in each block. Sequence reads for a sample are augmented with sequence reads from other identical by descent haplotypes. The method gives an approximate 40-50% reduction in genotype error rates over a competing method (Beagle v5.0) on an evaluation data set with 0.5x sequence coverage when compared to array genotypes. The HBImpute genotypes were used for association analysis and phenotype prediction, and yielded results that were similar to results obtained for SNP array data. The augmented sequence coverage appears to improve detection and calling of copy number variants. Software implementing the method is freely available for non-commercial use.

Comments

The type of genotype imputation should be clearly delineated in the introduction to avoid potential confusion. It appears that you are imputing sporadic missing genotypes, and not imputing missing markers using an external reference panel, or genotypes from genotype likelihoods. Is this correct. Would methods for imputing genotypes from genotype likelihoods be a better solution here?

The introduction was modified to clarify that our aim is on the imputation of sporadically missing genotypes and not the increase of the marker density (line 70 f.). The use of genotype likelihood is indeed a common input format used for other datasets, however, as we are using doubled haploid lines all genotyping calls should be homozygous. Because of this, likelihoods are usually basically 1 for homozygosity in one allele and otherwise typically indicate problems in the genotype calling like a duplication that led to reads from different areas of the genome to be mapped to this base pair. Thus, the additional information from genotype likelihood is far inferior to the software improvements between BEAGLE 4.1 and BEAGLE 5.0. Discussion on this has been added to both the results and the discussion section (lines 169 ff. & lines 306 ff.).

Software license restrictions should be noted in the paper.

As the HBimpute step is under a patent, the software license restriction should be covered by the Competing interests session (lines 656 ff.). Use in academia is possible without restrictions. More direct instructions on how to use the associated software package can be found on GitHub. We feel like stating this in the main part of the manuscript would be too much and thus decided to now more formally refer to both GitHub and the competing interests. Sadly, making the package available via CRAN is not really possible as a call to external software (BEAGLE) is not in line with the CRAN policies.

In the initial VCF file that is produced from low coverage sequence data, what thresholds determine whether a genotype is called or set the genotype to missing?

All non-default parameters in FreeBayes are now specified in the materials and methods section. In addition to that manual quality control filtering was applied (lines 428 ff.).

The term “cells” is used several times on p 4/17. Please define “cell” or use a different term.

We define a cell as an entry in the genotype matrix. We chose the wording cell to be consistent with the HaploBlocker publication (Pook et al. 2019). An explanation on this was added to the manuscript (lines 112 ff.).

In Table 1, what is the input data to Beagle for the results in the "Beagle" column? Is it the imputed data set that is used to generate the haplotype library?

The input dataset for BEAGLE is the raw SNP dataset that is obtained after the use of FreeBayes and quality control filtering. Thus, the output of BEAGLE is equivalent to the auxiliary imputed SNP dataset – a statement on this was added to the manuscript (lines 131 ff.).

P. 5/17, "observe an increased number of markers for all MAFs". Increased compared to what?

In the sentence, a statement like "compared to the 600k array" was missing. By adding STITCH and Beagle 4.1 to the analysis, the resulting allele frequency spectra are now much less similar and thus nicely highlight different types of errors occurring (e.g. loss of minor variants with STITCH, compared to an overrepresentation of minor variants with BEAGLE 4.1 (Figure 3). The entire paragraph has been rewritten (lines 199 ff.).

Figure 4 – What is varying to produce the each curve – presumably it is the significance threshold. This should be stated in the figure text. Also the left figure would be a bit easier to interpret if the order of lines in the legend was consistent with the order the lines in the left figure.

You are indeed correct. This figure was generated by calculating the number of identified QTL and the share of positives hits for various significances thresholds. The use of a single significance threshold is not applicable as p-values for higher marker densities naturally are higher and adaptation of thresholds to account for multiple testing is also not straightforward. Thus, this is the best solution we could see for a fair comparison between datasets (lines 257 ff.).

We do see reasons for ordering entries in the legend according to the order of the lines in the figure. However, we feel that ordering according to data type (first all array-based lines, HBimpute based, etc.) still is preferable, in particular, because the order between lines in the two subfigures is different. The color scheme in the Figure was adapted to use more similar colors for similar approaches.

In Methods, can you indicate how overlapping blocks (as in Figure 5) are handled?

Reads from all individuals that are locally in the same block are used. Thus, in case an individual carries multiple blocks, all reads from individuals in both blocks are used. Individuals that carry both blocks are not used twice. We extended the respective paragraph to clarify (lines 488 ff.).

P. 6/17, how is "predictive ability" defined?

The predictive ability is defined as the correlation between the phenotypes and the estimated breeding values in the test set without any correction for heritability. As this is fundamental to the results, we now also mention the formal definition in the Results section (lines 244 ff.).

Figure 5, please describe what the left and right sides represent in the figure text

The left side displays a dataset before imputation and the right side after imputation. We added headings to indicate that these datasets are examples for the raw SNP dataset and the HBimpute SNP dataset (now Figure 6). The figure caption was extended accordingly.

Line 358. Does the read coverage computed after running HB include up-weighted reads from individuals?

Thanks for spotting! The read-depth when only using reads from the individual itself is now set to only one times the individual read-depth as there is no reason to up-weight here. In case reads from more individuals are used, we still report the higher read depth to put more emphasis on the individual itself. A brief statement on the number of independent reads used in the genotype calling was added (lines 125 ff.).

“Markers above a certain p-value” (line 451). Do you mean “above” or “below”?

Thanks for spotting! Below is indeed correct (line 546).

Reviewer #2: Pook et al. presents a new pipeline, HBimpute, for imputation of low-coverage WGS data, designed for plant genetics. The key feature of the pipeline is to locally merge reads of different lines when they share a haplotype block, to increase the read depth of the genotype calling procedure, leading to better imputed genotypes compared to BEAGLEv5.

The pipeline presented in the manuscript improves the discordance rates of imputed sequence data when benchmarked against SNP array data and high coverage WGS, at a cost of an increased computational time.

I have few questions and comments regarding the manuscript and the benchmarking, which in some places seems to be lacking. In particular, the manuscript seems to focus on benchmarking the HBimpute pipeline against standard imputation (based on hard calls). However, due to the nature of low-coverage WGS, typically this type of data is imputed from genotype likelihoods, rather than hard calls. An evaluation of the HBimpute pipeline and methods specifically based for imputation of low-coverage WGS (BEAGLEv4.1 and STITCH) is therefore needed.

At least in our tests, the use of genotype likelihood in methods like BEAGLE 4.1 was not beneficial. Results were basically the same as when using BEAGLE 4.1 with gt parameter. We are assuming this is due to genotype likelihood not providing much additional information for doubled haploid lines – or actually even introducing noise by allowing for more non-homozygous calls which are oftentimes caused by genotyping errors. Furthermore, BEAGLE 4.1 has previously been shown to perform subpar for doubled haploid lines, which was much improved in later versions (lines 306 ff.).

We added an extended comparison to both BEAGLE 4.1 and STITCH, which further highlights the potential of our suggested pipeline. Nonetheless, STITCH provides highly accurate genotyping calls for those cells in the dataset that can be called, however, seems to have some weakness regarding the imputation / calling of rare variants (lines 311 ff.; Figure 2 & 3).

Additionally, other metrics to assess the quality of the genotype calls need to be provided.

We agree that just reporting the plain error rates was not sufficient as this puts a higher weighting on common variants. To complement this, we added an extended analysis on the performance of all considered imputation algorithms depending on the allele frequency of a given variant (lines 175 ff., Figure 2, Figure S1). As the use of correlations between underlying true genotypes and imputed genotypes shows very similar results to a plain comparison of error rates without information on the step they were called in, we feel like an extension in this direction is not need.

Major comments

1. The pipeline uses BEAGLEv5 to firstly impute missing data to derive a haplotype library with HaploBlocker. This is subsequently used to improve the quality of the raw SNP dataset, prior to a final imputation with BEAGLEv5. While I do understand the benefit of this, compared to imputation on the raw SNP dataset alone, I am not convinced that this is the best procedure.

Methods like BEAGLEv4.1 are able to use genotype likelihoods (obtained in your case with Freebayes) instead of hard-called genotypes to produce reference-aware genotype calls, that use information from all other target samples. As BEAGLEv4.1 outputs only positions having at least one read covered, missing likelihoods can be called as uniform, or standard imputation with BEAGLEv5 can be run afterwards, as performed in (Homburger et al., 2019).

The authors should show how their pipeline compares to genotype likelihoods with BEAGLEv4.1 (with uniform likelihoods to replace missing likelihoods) and BEAGLEv4.1+BEAGLEv5.0.

We added a comparison on the use of BEAGLE 4.1. To keep the main focus of the manuscript on our new imputation pipeline we decided to not include extended discussion on the use of BEAGLE 4.1 + BEAGLE 5.0 pipelines, as results for genotype likelihoods in BEAGLE 4.1 should already show the inferiority of the approach in the setting of fully inbred material.

2. As the authors work with low-coverage data where a small number of founders is known or can be derived, they should compare the imputation performance of their pipeline and the method STITCH (Davies et al, 2016), as the method seems to be well designed for exactly the same task.

An extended comparison between HBimpute and STITCH was added. For those cells in which STITCH is making a genotype call, the performance is actually slightly better than HB-array. However, STITCH seems to struggle to call rare variants. Thus, a high number of variants gets lost and overall error rates are higher than with our suggested pipelines (Figure 2).

We tested more complex pipelines that repeat the HBimpute step multiple times with auxiliary imputed SNP datasets derived in different ways – here the higher accuracy of STITCH on a share of the dataset led to slightly improved results (lines 315ff.). As the complexity of such a pipeline is substantially higher, separate VCFs and BAM files need to be processed, and computing times increase substantially, we decided to just briefly mention this as a potential extension in the discussion, but not overload the manuscript with the full extended pipeline.

3. The authors show discordance rates as the only metric for genotype accuracy. They should show how their pipeline performs for different type of variantion (e.g. SNPs vs indels), add both marker-level accuracy (such as Pearson correlation), and additional genotype-level accuracy, to show the difference between REF and ALT calls. Stratifying the accuracy by minor or non-reference allele frequency could also add value to the result section and to distinguish the different methods.

We added a figure displaying error rates depending on the allele frequency of the given marker (Figure 2; Figure S1). As correlations were mostly affected by the share of missingness in a given marker and the problem of underrepresentation of rare markers should be compensated by the additional figure, we decided against showing results regarding genotype accuracy based on correlation. We would generally expect higher error rates in indels, however, at least for the 6,759 indels on the 600k array, there were no significant differences. We decided to not report this in the manuscript as the chosen indels represent extremely high-quality variants (ascertainment bias) and are probably not really representative of indels in general.

4. All the figures should be drastically improved as often they are hard to read, lack of titles and axes descriptions.

The overall quality of all Figures should be improved in the new version with additional titles, axis descriptions, figure captions, and improve color schemes.

Minor comments

-- Would be interesting to know the impact of the local merging of reads on the reference bias. A quantification of this would add value to the manuscript.

By the use of HBimpute, the allele frequency spectrum is slightly tilted towards the most frequent variant in the given position, as the chance to be in a block that is including this variant is just the

highest. A figure on this was added to the Supplementary material (Figure S2). We would assume that all imputation algorithms should be tilted towards the most frequent allele – of the tested methods this effect was smallest in BEAGLE (Figure 3). As in particular BEAGLE 4.1 imputed datasets showed variants not present on the array, we would assume that some of this additional variance is actually due to imputing errors (lines 199 ff.).

--Page 6, line 170. (connected to point 3) The authors talk about increased power to call structural variation. This statement should be backed up showing the improvement obtained, by using high coverage data as a validation.

At this stage, the results regarding the detection of structural variation should be mostly seen as a proof of concept. Both the reported correlations (lines 221 ff.) and Figure 4 illustrate the potential of the method as this does contain a comparison to high coverage data (30X). However, as the read-depth in the final dataset is not dependent on the sequencing depth but the number of lines in local IBD, existing software for CNV calling is just not applicable as the local read-depth is depending on the number of lines in a local haplotype block and not the sequencing depth (lines 410 ff.). Statements have been weakened to make sure that structural variation in the proof of concept should only be seen as copy number variation.

This is definitely something that we plan to work on in the near future.

--Page 6, line 180, Figure 3B/D. Assuming that Fig B is the high coverage (not very clear), the authors could also explain why there is a peak of read depth in the tail of Fig D.

--Page 6, line 192. (connected to point 3) The authors should validate the CNV calls you derived from the HBimpute pipeline using the 30x data to explain the results of Table 2.

We added further labels to the Figure to clarify which plot is displaying what. The peak in the tail of Figure 4.D. is a false-positive CNV call in the HBimpute pipeline (assuming the 30X data to be correct). Imputation still is not perfect (correlation ~0.75), however, in particular the total number of false positives is substantially lower than when using the plain 0.5X data. A plain comparison on the overlap between areas with read-depth above 1.5X was added and also showed a significantly higher share of overlap between 30X and HBimpute 0.5X than 30X and raw 0.5X (lines 230 ff.).

Nonetheless, we still have to note that calling CNVs from HBimpute at this stage should only be seen as a proof of concept. Not more, but also not less.

-- Page 7, line 196: This section is hard to follow and might be restructured. I am not sure what are and how strong are the conclusions of this GWAS.

The GWAS analysis was redone using the statgenGWAS package that uses method suggested by Kang et al. 2010. This approach has shown to be much more powerful for our data than the methods used before (Yu et al. 2006). The variance of results has been further reduced by more repetition /

simulated traits. Results of all sequence data panels (except BEAGLE 4.1 imputation) are now showing the better performance of the sequence data compared to array data (Figure 5.A).

In terms of fine-mapping, results of the array data are still on a similar level than for the sequence data – which might not be a possible result for the use of sequence data, but we still feel is of interest for potential readers (lines 292 ff.).

-- Page 7, line 206: What filters have been used prior to the GWAS?

No additional filtering was applied, as quality control filtering was already extensively performed in the imputation procedure (line 586).

-- Page 7, line 206: authors should elaborate more on the fact that plain BEAGLE imputation gets better results in the GWAS setting than HB-seq, even though it does have bigger discordance rates

The biggest reasons, we see for this are slightly more single marker peaks due to mapping problems (lines 279 ff.) in HB-seq imputation compared to BEAGLE 5.0 and sampling effects as differences are minimal.

In our new test that is using a more suited GWAS method (Kang et al 2010) with an increased number of simulated traits to reduce variance, HB-seq now leads to slightly better results than BEAGLE 5.0 – practically speaking, we would however still classify the results to both datasets to be basically of the same quality. As both GWAS and genomic prediction are whole-genome analyses and differences in imputation accuracy of 0.35% are not really that impactful – we would assume the effect to be higher when analyzing specific areas of the genome more deeply. We decided to nonetheless stick with these "standard" applications as the main focus of the manuscript is intended to be on the imputing pipeline and not the downstream analysis.

-- Page 9, line 301: with 0.5x coverage, it it relatively unlikely to get 5 observations. The authors might want to decrease the threshold, and check if the performance of BEAGLE5 increases.

The use of at least five supporting observations was done to ensure high data quality as substantially increased error rates in all imputation pipelines and worse performance in the downstream applications were observed when decreasing this threshold. As the main topic of the manuscript is intended to be on the imputation pipeline and not general filtering criteria for sequence data, we feel that going into more detail would go beyond the scope of the manuscript.

-- Page 9, line 339: Parameters and reference panels used for BEAGLE are not clear to me.

Regarding the use in the HBimpute step: The haplotype library is calculated for the entire dataset containing both the dataset itself and the reference panel. Thus, the downstream read-merging can also consider reads from these individuals.

Furthermore, additional lines can provide information in any BEAGLE imputation, even when only performing imputation of sporadically missing markers.

We added a reference to a paper on the adaption of parameter settings in BEAGLE for crop datasets and now more explicitly report the BEAGLE parameter settings used to obtain all reported results (lines 142 f.).

--Page 11, line 407. There might be a typo regarding the read depth of marker 4

As far as we can tell, this sentence did not contain a typo. Nonetheless, we decided to rewrite the sentence to make the statement clearer (lines 539 ff.).

-- Page 11, line 424: Not sure I agree with the statement: "Phasing accuracy of low read-depth sequence data is usually relatively low". Intuitively, I would think that phasing accuracy (for >0.5x data) decreases increasing the coverage, as the number rare variants to be phased also increases, and these are very hard to phase. An explanation would be useful.

When comparing to genotype data obtained with a chip or using long-read sequencing our statement should still hold true – assuming the marker density stays the same (with more markers there is naturally more opportunity to make phasing errors). Note that for HaploBlocker the physical distance between phasing errors is of importance. Overall, sequencing using short reads will probably always struggle with phasing unless when using high sequencing depth on the individuals themselves or at least one of the parents. The paragraph has been extended to clarify (lines 558 ff.).

--Page 12, line 480. Computational resources used to run BEAGLE (running time/memory usage) is missing.

Information on the memory usage of HBimpute was added to the manuscript. Note that the main bottlenecks are due to other software/packages (BEAGLE & HaploBlocker), thus a detailed discussion is omitted here. Information on the other approaches considered was also added (lines 623 ff.). Note that for all approaches preprocessing (alignment etc.) will take substantially more computational resources.

Reviewer #3: In this manuscript, Pook and colleagues describe a new imputation pipeline for calling genotypes from whole-genome sequence data with very low read depth (0.5X). A major goal of the pipeline is to increase marker density though genotype-by-sequencing (GBS) when compared to genotype arrays, for the purposes of e.g. genomic prediction or association studies. Using sequence data from doubled haploid maize lines, the authors demonstrate lower imputation error rates with

HBimpute when compared to BEAGLE. When these imputed markers are used for genomic prediction, the results are somewhat mixed—there is a reduction in predictive ability that the authors claim is due to the poorer quality of additional markers gained from the GBS approach.

While I do not doubt the pipeline by the authors produced lower imputation error in their dataset when compared to BEAGLE, I have concerns about some of the authors' claims as well as with the novelty and broader applicability of the approach.

Average read depth is a useful shorthand to communicate the rigor of sequencing efforts across different platforms and pipelines. Here, the authors merge reads to claim a "virtual read depth" of 83X. This would be fine if they were interested in variants from a population or a species, but is definitely misleading when they are interested in the genotypes from specific lines and individuals. The claim that this depth of coverage enables structural variation (only CNVs) to be called is similarly misleading because they are called on what is effectively a population level when the method is aimed at describing genotypes at the individual level. I think the manuscript would be improved if the distinction between describing variation at the individual and the population level was made clearer.

While it is true that the local read-depth of different lines that are locally in the same haplotype block is usually rather similar, there are still within-population differences. The share of allowed differences between two lines that are put in a joint haplotype block can be directly controlled via parameter settings from the HaploBlocker package. Thus, the user can directly control if he is mostly interested in CNVs at the population level and merge virtually all lines of a population or go for within-population differences. A short discussion of this was added to the manuscript (lines 415 ff.) Note that the genomic dataset used here was derived from a single European maize landrace and still showed substantial differences between the individual lines.

Statements regarding structural variation were put into context and clarification that at this stage this only includes CNVs was added (lines 406 ff. & 523ff.).

The authors cite several approaches for imputation, but only benchmark their pipeline to BEAGLE and only with their dataset of doubled haploid maize lines. A custom pipeline for a specific dataset will typically produce better results than one off the shelf. To convincingly demonstrate better performance, the pipeline should be benchmarked against at least more than one existing method. Limitations from data availability may preclude the authors from testing the approach on additional datasets, but they should consider simulation strategies. Broadening the applicability of their approach would increase the appeal of the method to a wider audience.

We added an extended comparison between our approach, BEAGLE 4.1 and STITCH. Regarding other datasets, we did tests with datasets containing heterozygous material (chicken). However, as short-read sequencing was used, phasing still remains a major issue and performance is "only" on par with BEAGLE 5.0. As we would not recommend the use of HBimpute on short-read sequencing data in heterozygous material at this point, we decided to not include this analysis in the manuscript.

Finally, while the goal of the new pipeline is to increase the number of available markers through GBS, array data is still treated as their gold standard. The error rates for imputed sequence are all reported as discordance with the genotyping array and genomic prediction is actually worse with the greater number of markers. The results would seem to indicate that array data is typically preferable than low depth whole-genome sequencing for the purposes that the authors are interested in. Heterozygote calls, for example, cannot be used from low depth sequencing while they are fine from array data.

The results for downstream applications like genomic prediction and GWAS should be highly linked to how well the data is able to estimate relatedness between individuals. Thus, with similar data quality in all markers, we would expect the performance of methods to increase (or at least stay the same if all required information is provided).

However, as the accuracy of genomic prediction is going down – this can/should be seen as an indication of lower data quality. We do see two main factors for this: added markers are genotyped/imputed with lower accuracy and additional markers are potentially in less important regions and thus ascertainment markers in the array construction are just more informative. Note that by reducing the marker set for the sequence data to just the array markers, the predictive ability is actually slightly higher can the use of array data. This comparison is not affected by sampling effects (which markers are included) but just reliant on the actual data quality and thus shows clear evidence that the overall data quality of sequence data is higher or at least on par with array data.

As we are working on inbred material, we would actually suggest to not use heterozygotes calls from both low read-depth sequencing and array data (although we of course agree that arrays would be more suitable for typing heterozygotes variants in not-inbred material).

Note that low read-depth sequence data is a cost-competitive genotyping approach to high-density array data. In particular for genomic prediction, we would however agree that array genotyping (if an array is available for the species) with a lower density should be the most cost-effective strategy. Extended analysis using down-sampled array data (10k & 50k) was added to the genomic prediction section. The discussion was extended accordingly (lines 337 ff.)

In our GWAS, we do see improvements of the use of sequence data over array data. However, we again observe that the marker density should not be increase too heavily as the lower data quality then will negate the positive effects of additional information (lines 285 ff.).

Minor:

The authors rightly indicate that additional SNPs artificially inflate the power of GWAS, as measured by p-value, in ways that are difficult to compare. However, they proceed to simulate QTL and compare the proportion of true positive hits and use this as a metric of performance. It is not obvious to me how this strategy produces a comparable metric and this section could be explained a little better.

Obtained QTLs from a set p-value from different datasets are not comparable and we are not aware of a 100% fair way to correct for this (p-values for larger datasets are typically smaller). The line for a given dataset represents all potential outcomes of a GWAS analysis with different values representing different p-values. This allows the comparison of results of GWAS studies with different

significances thresholds (e.g., how many of the top 3 GWAS hits are actual underlying QTLs). Similarly, an entry with more identified real QTLs and a higher share of true positive calls will represent a case of strict improvement. Explanation on this was also added to the manuscript (lines 266 ff.).

L193:

negligible should probably be used here and throughout the manuscript instead of “neglectable”

Done. Thanks! (lines 48, 166, 606)

Have all data underlying the figures and results presented in the manuscript been provided?

Large-scale datasets should be made available via a public repository as described in the PLOS Genetics data availability policy, and numerical data that underlies graphs or summary statistics should be provided in spreadsheet form as supporting information.

Reviewer #1: Yes

Reviewer #2: Yes

Reviewer #3: No: As stated by the authors, only some of the data has been made available due to its private ownership by KWS Saat.

Only genomic data for chromosome 1 was made publicly available. As the focus of the manuscript is on an imputation method, this should still be enough to validate the performance of our new imputing approach.
