# Supplementary material for: Increasing calling accuracy, coverage, and read-depth in sequence data by the use of haplotype blocks
Source: PLoS Genet. 2021 Dec 23;17(12):e1009944. doi: 10.1371/journal.pgen.1009944 (PMC8699914; doi:10.1371/journal.pgen.1009944)
Supplement: Attachment — Submitted filename: response_to_review_hbimpute3.pdf [file pgen.1009944.s006.pdf]

Dear PLoS Genetics Editorial Board, Dear reviewers,

Below you can find our responses to the comments made in the last round of review. For an exact overview of all changes made to the manuscript, we refer to the LatexDiff file (Revised Article with Changes Highlighted).

During resubmission, an additional problem broad up by the Editorial office was our Data availability statement, as we are only uploading genomic data for chromosome 1. As discussed in an earlier round of review, we feel that this does not impact reproducibility of results, as the methods works very similar on the different chromosomes and thus results across chromosomes are very very similar. Thus, we requested an exception for our case.

Note that we have submitted a request at our private partner to allow us to make data for all chromosomes available. As this can take a while and we did not want to hold up the peer-review process, we decided to resubmit without knowing the results of this request and hope for an exception from PLoS (but will of course make data available if we are allowed).

Best regards

Torsten Pook and co-authors

Reviewer #1: This is the revision of a manuscript presenting a method called HBImpute for estimating genotypes from low coverage (e.g. 0.5x) sequence data. The method is designed for the special case of samples with homozygous genotypes (double haploid lines) that arise in plant breeding. The method identifies haplotype blocks and clusters sequence reads from identical by descent haplotypes in each block. Sequence reads for a sample are augmented with sequence reads from other identical by descent haplotypes. The method gives an approximate 50% reduction in genotype error rates over a competing methods on an evaluation data set with 0.5x sequence coverage when compared to array genotypes. The HBImpute genotypes were used for association analysis and phenotype prediction, and yielded results that were similar to results obtained from SNP array data. The augmented sequence coverage appears to improve detection and calling of copy number variants.

The authors have addressed my previous comments. I have only two additional comments:

1) The response to the reviewers states that the software is freely available for academic research ("Use in academia is possible without restrictions"). This should also be stated in the published manuscript.

A corresponding statement has been added to the competing interests (lines 681f.). Thanks for the suggestion.

2) Line 479, "1.000 SNPs". Do you mean 1000 or 1?

This indeed was a typo and should be 1'000 (line 491).

Reviewer #2: Thank you for your response to the reviewer comments. The quality of the manuscript improved by introducing other methods to the benchmark.

However, I still find the manuscript lacking of important information. In particular the accuracy comparison against STITCH, the second best method after the HB pipeline, should be more broadly expanded, to justify and show where the benefit of the HB pipeline resides over STITCH that is at least 5 times more computationally efficient then HB-array and 10 times more efficient than HB-seq (in both reported running time and memory), especially after seeing that they both show almost identical predictive power.

Although computing times for the HBimpute pipeline are higher than for STITCH, these times should still allow for application, and computing times in the pre-processing should typically be higher. A statement on this was added to the manuscript (lines 644ff.).

Although the performance of HBimpute and STITCH in regard to prediction and GWAS is similar, STITCH shows lower overall imputing accuracy for rare variants and a strong bias in the allele frequency spectrum as in particular rare variants tend to get lost. This may not affect prediction, but can be highly relevant in other analyses.

Major comments:

1. I appreciate the introduction of Figure 2. However, I honestly do not understand the author's comment about avoiding to use the well-known and standard (dosage) imputation r^2. Verifying not only hard calls, but also dosages, is important when imputation is performed. A quantification of error rates stratified by REF and ALT calls for all the methods, would also be useful.

Numbers regarding the accuracy of the imputation in terms of correlation and error rate for REF and ALT alleles have been added to Table 1 & 2 (lines 205ff.). We decided against an extended analysis of ALT/REF error rates as these differences seem to be mostly caused by differences in the initial calling from the array and high read-depth sequencing data (lines 182ff.).

2. Connected to the previous comment, calibration of genotype posteriors for the HB pipeline seems also easy to check and important to verify that the introduction of the haplotype block (and therefore the merging of the reads) is sound.

We are not sure what is meant here as HBimpute is only providing hard calls. The calculation of a suitable haplotype library is definitely mandatory for the use of HBimpute. We would expect that a low-quality haplotype library would result in basically no additional genotype calls from the HBimpute step and thus an imputed panel similar to plain use of BEAGLE 5.0 (or whatever additional software was used for the calculation of the auxiliary and final imputing step).

3. The repeated statement of having a "read depth of 83X" is very misleading. To my understanding, this inflation of read depth that the authors claim is "artificial" is just for internal use of the pipeline and it is not seen (to that amount) in practice. The method is doing slightly better than other methods that work on pure 0.5x data, showing that the reported 83x is an inflated estimate.

We feel like stating this high read-depth is not misleading as, on average, 81 different reads are used for the variant calling of each individual. Explanation on the context of read-depth in HBimpute was extended (lines 117ff.). Furthermore, we would argue that a reduction of the discordance rate from 0.89% to 0.47% is more than a slight improvement – in particular when considering that discordance rates between array data and 30X high read-depth sequence data for our lines were 0.30%, which could be seen as a lower limit for achievable error rates. A statement on discordance rates between array and sequence data was added to the results section (lines 182ff.).

4. The GWAS analyses is to me not convincing. The low sample size and no application of filtering (e.g. on MAF), lead to a questionable power and a small amount of error and bias in the genotype calls can produce many false positives.

The sample size of the data is definitely relatively small compared to large-scale human or animal studies but still corresponds to the size of common GWAS studies in plant breeding. We chose not to apply MAF filtering as the allele frequency spectrum is quite different between the imputation approaches, which in turn could lead to bias between datasets. To avoid problems of questionable significance, QTLs were only assigned to markers with a minimum minor allele frequency of 0.1. This is now also explicitly stated in the manuscript (lines 601ff.)

5. Authors need provide the parameters they used to run all methods. The manuscript needs to significantly improve in terms of reproducibility.

The script used for the GWAS analysis, GP and calculation of imputation error rates have been uploaded to GitHub to ensure full reproducibility of all results (lines 671f.). All non-default parameters used in STITCH, BEAGLE, BWA MEM, FreeBayes, and HaploBlocker are explicitly mentioned in the manuscript (lines 142f., 439ff., 485f.).

Reviewer #3: The revisions by Pook et al. have substantially improved the manuscript. I appreciate the additional comparison to STITCH, the expanded discussion, and I now find the article suitable for publication.
